# Purpura Hemorrhagica

**Published:** 1894-12

**Authors:** 


NOT long since a vicinity news correspondent of a Rochester paper reported a case of "bled-to-death from tooth extraction," and thus set the fidgety patient by the ears. Hoping for an item we wrote an esteemed member of the VIIth District Society, resident in the village near which the case had been located, and got for answer the following:

"In regard to the so-called 'bled-to-death' case, I have made inquiry of the attending physician and he gives me the following facts. This case was a pure and simple case of 'Purpura Hemorrhagica,' and had no particular dental relation other than that he bled from the mucous membrane of the mouth. He also bled from the nose and likewise from the bowels, but his final hemorrhage was in the brain. His physician tells me it was a constitutional case; that he had been called to see another patient and incidentally expressed the thought that the young man did not look well. He examined him and diagnosed the case at that time, putting him under treatment, but had little hopes from the first. This is all. It has no dental significance."

All of which goes to spoil another story.—*Odontographic Journal.*

